# Detection of rare variants among nuclei populating the arbuscular mycorrhizal fungal model species *Rhizophagus irregularis* DAOM197198

**DOI:** 10.1093/g3journal/jkae074

**Published:** 2024-04-24

**Authors:** David Manyara, Marisol Sánchez-García, Merce Montoliu-Nerin, Anna Rosling

**Affiliations:** Department of Ecology and Genetics, Uppsala University, Uppsala 752 36, Sweden; Department of Ecology and Genetics, Uppsala University, Uppsala 752 36, Sweden; Uppsala Biocentre, Department of Forest Mycology and Plant Pathology, Swedish University of Agricultural Sciences, Uppsala 750 07, Sweden; Department of Ecology and Genetics, Uppsala University, Uppsala 752 36, Sweden; Centro de Biotecnología y Genómica de Plantas, Universidad Politécnica de Madrid (UPM), Madrid 28223, Spain; Department of Ecology and Genetics, Uppsala University, Uppsala 752 36, Sweden

**Keywords:** AM fungi, pooled spores and mycelia, pooled samples, single nuclei, single spore, low-frequency variants, SNP calling, intraorganismal variation

## Abstract

Identifying genuine polymorphic variants is a significant challenge in sequence data analysis, although detecting low-frequency variants in sequence data is essential for estimating demographic parameters and investigating genetic processes, such as selection, within populations. Arbuscular mycorrhizal (AM) fungi are multinucleate organisms, in which individual nuclei collectively operate as a population, and the extent of genetic variation across nuclei has long been an area of scientific interest. In this study, we investigated the patterns of polymorphism discovery and the alternate allele frequency distribution by comparing polymorphism discovery in 2 distinct genomic sequence datasets of the AM fungus model species, *Rhizophagus irregularis* strain DAOM197198. The 2 datasets used in this study are publicly available and were generated either from pooled spores and hyphae or amplified single nuclei from a single spore. We also estimated the intraorganismal variation within the DAOM197198 strain. Our results showed that the 2 datasets exhibited different frequency patterns for discovered variants. The whole-organism dataset showed a distribution spanning low-, intermediate-, and high-frequency variants, whereas the single-nucleus dataset predominantly featured low-frequency variants with smaller proportions in intermediate and high frequencies. Furthermore, single nucleotide polymorphism density estimates within both the whole organism and individual nuclei confirmed the low intraorganismal variation of the DAOM197198 strain and that most variants are rare. Our study highlights the methodological challenges associated with detecting low-frequency variants in AM fungal whole-genome sequence data and demonstrates that alternate alleles can be reliably identified in single nuclei of AM fungi.

## Introduction

Virtually all terrestrial plants depend on symbiotic interactions with fungi (Radhakrishnan *et al*. 2020). The arbuscular mycorrhizal (AM) fungal lineage evolved over 450 million years ago and is together with fine endophytes in Mucoromycota, thought to have been instrumental in the emergence of terrestrial plants (Redecker *et al*. 2000; Brundrett 2002; Hoysted *et al*. 2019). AM fungi are obligate plant symbionts that primarily sporulate underground and form mutualistic associations with the roots of a wide range of plant species in natural and agricultural systems. These fungi obtain carbon and energy from their colonized host roots and, in return, aid the host plants in accessing essential nutrients (phosphorus, sulfur, and nitrogen), and micronutrients, and facilitate the uptake of water from the soil (Smith and Read 2008; Bonfante and Anca 2009). By mediating nutrient uptake and sequestering carbon in the soil, the mutualistic symbiosis between AM fungi and plants provides core functions to terrestrial ecosystems (van der Heijden *et al*. 1998; Asghari *et al*. 2005; Smith and Smith 2011). Although AM fungi are ubiquitous in terrestrial ecosystems, their species diversity is relatively limited, with only approximately 350 species currently morphologically characterized and formally described (amf-phylogeny.com). However, molecular detection from soil and other environmental samples suggests that the actual number of species, estimated as virtual taxa based on DNA sequence data, could be much greater, potentially reaching up to 1,600 species (Öpik and Davison 2016).

Across the fungal kingdom, AM fungi are biologically and evolutionarily remarkable due to a unique combination of features. They grow primarily with aseptate hyphae and propagate with spores harboring hundreds to thousands of nuclei. Their nuclei migrate and mix within the shared cytoplasm of the organism (Jany and Pawlowska 2010; Marleau *et al*. 2011). Unlike other fungi with multinucleate hyphae and spores, no life stage with only 1 or 2 nuclei is yet known in AM fungi (Sanders and Croll 2010; Kokkoris *et al*. 2020). Drawing from microscopy observations of nucleus behavior in AM fungal mycelia and spores (Jany and Pawlowska 2010; Marleau *et al*. 2011; Kokkoris *et al*. 2020), we concur that genomes of AM fungal strains are best understood as populations of asexually reproducing haploid nuclei (Manley *et al*. 2023; van Creij *et al*. 2023). In the absence of observed sexual reproduction, a distinct single-nucleus stage, and strong evidence for recombination, mutations are anticipated to accumulate in AM fungi, leading to high genetic variation. However, studies have revealed that the overall genetic variation, measured by the density of single nucleotide polymorphisms (SNPs) within the genome, is relatively low in AM fungi, ranging from 0.23 to 0.43 SNPs/kb (Tisserant *et al*. 2013; Lin *et al*. 2014; Chen, Mathieu, *et al*. 2018; Morin *et al*. 2019; Sahraei *et al*. 2022). In contrast, true heterokaryotic fungal strains have been reported to have higher densities of SNPs, ranging between 15.9 and 23.7 SNPs/kb as reviewed in van Creij *et al*. (2020). Genetic variation in AM fungal strains has been extensively studied using sequence data generated through whole-genome sequencing of pooled spores and hyphae (Tisserant *et al*. 2013; Chen, Morin, *et al*. 2018; Morin *et al*. 2019; Sun *et al*. 2019) or whole-genome amplification and sequencing of individual nuclei (Lin *et al*. 2014; Chen, Mathieu, *et al*. 2018; Sahraei *et al*. 2022). Whole-genome sequencing of pooled spores and hyphae produced the first reference genome of the model strain DAOM197198 of *Rhizophagus irregularis* (Tisserant *et al*. 2013) and has revealed low levels of genetic variation in *R. irregularis*, *Rhizophagus cerebriforme*, *Rhizophagus diaphanus*, and other AM fungal genera such as *Gigaspora* (Morin *et al*. 2019) and *Diversispora* (Sun *et al*. 2019). Observations of low levels of genetic variation have been further supported by whole-genome amplification and sequencing of individual nuclei from several strains of *R. irregularis* (Lin *et al*. 2014; Chen, Morin, *et al*. 2018) as well as from cultures of *Funneliformis mosseae* and spores of *Funneliformis geosporum* collected from the field (Sahraei *et al*. 2022). A first step toward exploring mechanisms behind the observed low levels of genetic variation is to accurately map the distribution of rare alleles across nuclei.

The estimation of intraorganismal genetic variation relies on the accurate detection and analysis of SNPs obtained by mapping reads to a reference genome. However, the use of sequencing data from either pooled or individually amplified samples to uncover assayable genetic variation is associated with intrinsic biological and methodological biases. As highlighted in studies of population genetics, the pooling of samples can potentially lead to the loss of important biological information related to individual haplotypes and heterozygosity and poses the risk of missing rare alleles, particularly when a large number of individuals are pooled together (Cutler and Jensen 2010). Due to large variations in read depth within samples and incomplete coverage across the genome, it has been suggested that AM fungal sequence data obtained from amplified individual nuclei may offer lower resolution when determining quantitative variation at multiple loci among different lines (Robbins *et al*. 2021). Furthermore, errors stemming from the sequencing process and the amplification step with multiple displacement amplification (MDA) can complicate the detection of true biological signals (Rodrigue *et al*. 2009; Ropars and Corradi 2015). Previous studies investigating genetic variation in AM fungi have utilized various variant calling and filtering techniques to optimize the reliable detection of polymorphisms. Many of these approaches rely on standard methods based on predefined genetic and population models, especially the user-specified ploidy parameter, implemented through the haploid (Chen, Mathieu, *et al*. 2018; Chen, Morin, *et al*. 2018; Sahraei *et al*. 2022; van Creij *et al*. 2023) or the diploid (Ropars *et al*. 2016; Morin *et al*. 2019) options in variant calling algorithms. However, it has been pointed out that applying such predefined genetic and population models poses undesirable constraints on variant calling in AM fungal genomes, composed of a population of haploid nuclei potentially resulting in multiple alleles per locus and allele frequencies spanning the entire range from 0 to 100% (Wyss *et al*. 2016; Masclaux *et al*. 2019; van Creij *et al*. 2023). Thus, assumptions of the data and the methods used for variant detection significantly impact the detection of intraorganismal polymorphisms, with a notable bias against low-frequency variants. With the estimated number of nuclei in the AM fungal spore reaching up to 35,000 (Kokkoris *et al*. 2020), several previous studies have accounted for the potentially heterogeneous population of nuclei coexisting within an organism in *R. irregularis* strains by implementing a high ploidy level setting of 10 during variant calling (Wyss *et al*. 2016; Savary *et al*. 2018; Masclaux *et al*. 2019). This approach enabled the detection of both low-frequency alleles and multiallelic sites, many of which were recovered and confirmed in gene expression data (Wyss *et al*. 2016; Savary *et al*. 2018; Masclaux *et al*. 2019). Implementing high ploidy level parameters during variant calling has also been applied in other organisms including polyploid plant species like rye (Hawliczek *et al*. 2020) and in protists (Majda *et al*. 2019). Indeed, the detection of low-frequency variants is crucial for estimating demographic parameters and investigating genetic processes, such as selection, within populations (Lynch *et al*. 2016). However, the detection of low-frequency variants has to be cautiously conducted to ensure the accurate capture of true biological signals while effectively distinguishing them from potential errors.

In this study, we investigated intraorganismal genetic variation and explored the detection and frequency distribution of variants within the DAOM197198 strain of the model AM fungi *R. irregularis*. For this, we utilized the recently published reference genome assemblies of *R. irregularis* DAOM197198 (Manley *et al*. 2023) along with published whole-genome sequence datasets generated from amplified single nuclei (Montoliu-Nerin *et al*. 2021) and from pooled spores and mycelia grown in axenic culture (Chen, Morin, *et al*. 2018). Previous studies have shown that the DAOM197198 genome is haploid, homokaryotic for the *mat*-locus, and has low levels of nucleotide polymorphism (0.23–0.43 SNPs/kb) (Tisserant *et al*. 2013; Lin *et al*. 2014; Morin *et al*. 2019). Drawing upon the model that nuclei in AM fungi function as populations of asexually reproducing units (Jany and Pawlowska 2010; Marleau *et al*. 2011; Kokkoris *et al*. 2020; Sperschneider *et al*. 2023), we conducted variant calling across nuclei and within the whole organism to analyze overlapping and diverging variants detected in the 2 datasets. This allowed us to assess methodological limitations in detecting rare variants and to estimate SNP density per nucleus.

## Materials and methods

### Genomic data

In the study by Montoliu-Nerin *et al*. (2021), amplified single-nucleus genome data for the AM fungal model species *R. irregularis* strain DAOM197198 were generated from a single spore obtained from the Department of Agriculture and Agri-food Canada (AAFC), Government of Canada. In brief, a spore was isolated and crushed in 1× PBS and stained with SYBR Green I Nucleic acid stain before fluorescence-activated cell sorting of single nuclei. Individual nuclei were whole genome amplified by MDA, and, after screening for fungal DNA, 24 nucleus samples were selected and separately sequenced using Illumina HiSeq-X at the SNP&SEQ Technology Platform in Uppsala, at the National Genomics Infrastructure Sweden and Science for Life Laboratory (Montoliu-Nerin *et al*. 2021, 2020). Throughout this paper, we refer to this dataset as the single-nucleus dataset.

In the study by Chen, Morin, *et al*. (2018), whole-genome data were generated from samples representing a pool of spores and mycelia of the same strain DAOM197198, maintained in axenic conditions at Agronutrition (Labège, France). The samples were sequenced using a paired-end (2 × 125 bp) TruSeq Nano library for Illumina, and the sequencing was performed on an Illumina HiSeq 2500 platform and used for the *R. irregularis* DAOM197198 version 2.1 reference genome assembly (Chen, Morin, *et al*. 2018). The data were provided by Francis Martin (INRA, France). Throughout this paper, we refer to this dataset as the whole-organism dataset.

### Read mapping

Sets of paired-end reads from 24 single nuclei and 1 set of paired-end reads from whole organism were mapped to the chromosome-level genome assembly of DAOM197198 (Manley *et al*. 2023) (see Supplementary Table 1) using the BWA-MEM algorithm of Burrows–Wheeler aligner (BWA) v.0.7.15 (Li *et al*. 2009). To analyze the impact of the reference assembly on SNP calling, the whole-organism dataset was also mapped to 3 additional genome assemblies (Chen, Morin, *et al*. 2018; Montoliu-Nerin *et al*. 2021; Yildirir *et al*. 2022) (see Supplementary Methods and Results for details; see Supplementary Table 1). However, for all other analyses herein, only the most recent chromosome-level genome assembly with 32 complete and contiguous chromosomes (Manley *et al*. 2023) was utilized. The resulting Sequence Alignment Map (SAM) files were then converted into Binary Alignment Map (BAM) files using SAMtools v.1.5 (Li *et al*. 2009). Picard v.2.10.3 (http://broadinstitute.github.io/picard/) was used to identify and tag duplicate reads in the BAM files, sort the BAM files by coordinates, and replace read groups. To evaluate the completeness of the single-nucleus dataset, 2 rounds of mapping with the single-nucleus data to the reference genome were performed. First, the 24 sets of paired-end reads were individually mapped to the genome assembly and, second, the 24 sets were then merged into a single dataset and collectively mapped to the genome assembly (see Supplementary Tables 2 and 3). Only the 24 separate sets that were individually mapped were used for downstream analysis of variance.

To assess the read quality and data completeness in both the whole-organism and the 24 amplified single-nucleus samples, the percentage of reads mapped and the percentage of the reference genome covered by at least 1× and 5× read depths were computed using Qualimap v.2.2.1 (Okonechnikov *et al*. 2016). Based on this analysis, we excluded 3 nuclei (nucleus 2, 12, and 23) from downstream analyses because they had the lowest percentages of the reference genome coverage at 1× and 5× read depths (see Supplementary Table 2). The average depth of reads across the reference genome was computed using mosdepth (Pedersen and Quinlan 2018).

### Variant calling

Genetic variation was assessed by analyzing SNPs through variant calling on mapped reads using Freebayes v.1.3.2 (Garrison and Marth 2012). Freebayes is a versatile tool and can be used on genomes of any ploidy, pooled samples, or mixed populations. To evaluate the effect of ploidy settings on SNP calling and alternate allele frequency (AAF) distribution, the different ploidy settings 1, 2, 5, 10, 15, and 20 were tested in combination with variant calling parameters -O (standard filters) and -u (remove complex allele observations from input), using the whole-organism dataset mapped to the reference assembly (Manley *et al*. 2023) (see Supplementary Methods and Results for details; see Supplementary Table 4). The conceptual hypothesis that the genome of *R. irregularis* is manifested as a population of independently replicating and evolving nuclei suggests that variants may be present at low frequencies when carried only by a small fraction of all nuclei. Based on our ploidy setting analysis, the ploidy level 10 setting was applied in all subsequent SNP calling with the abovementioned parameters. We concluded that this setting accommodated the biology of our study organism by ensuring that variant detection, particularly low-frequency variants, is not limited by ploidy settings.

In both datasets, called SNPs were filtered using vcffilter from the vcf-lib (Garrison *et al*. 2022) and BCFtools v.1.12 (Danecek and McCarthy 2017) to retain only polymorphic SNPs with a phred-scaled quality score of at least 30 (QUAL > 30). The SelectVariants tool from the Genome Analysis Toolkit (GATK) v.4.2.5.0 (McKenna *et al*. 2010) was used to subset SNPs in both the coding sequence (CDS) and the nonrepetitive regions of the reference genome. A small fraction of the SNPs were multiallelic, and these were excluded from downstream analysis (see Supplementary Table 5). Next, filters to include only high-quality SNPs and to minimize false positives were implemented to account for the biological and technical constraints of the 2 datasets. For this, only biallelic sites with a minimum of 5 reads supporting the alternate allele were retained in the whole-organism dataset. In the single-nucleus dataset, biallelic sites were hard filtered to identify polymorphism across nuclei by requiring genotypes in each haploid nucleus to be covered by a minimum of 5 reads and have an alternate allele fraction of at least 0.9 to be scored as an alternate allele and equal to or below 0.1 to be scored as a reference allele. Within individual nuclei, genotypes that did not meet these criteria were masked as missing data. The filter for allele fraction within nuclei was designed to account for haploid nuclei by excluding sites that were polymorphic within individual nuclei potentially resulting from errors introduced during the MDA process, sequencing errors, or mismapped reads. Finally, population-level hard filtering was applied to retain only sites that were polymorphic across nuclei and supported by data from at least 67% of the analyzed nucleus samples. The AAF was calculated as the proportion of nuclei supporting the alternate allele out of all nuclei with data at the site. In the whole-organism dataset, on the other hand, AAFs were calculated by dividing the number of reads supporting the alternate allele by the total number of reads at the site. The vcfR v.1.14.0 R package (Knaus and Grünwald 2017) was used to calculate AAFs that were then visualized through histograms generated with the ggplot2 package within R v.4.1.3 (R Core Team 2021).

Our ability to detect SNPs in the single-nucleus dataset encompassing 21 individual nucleus samples was constrained by only partial coverage of the reference genome by each of the nucleus samples. Consequently, the number of potential SNPs supported by data from at least 67% of the 21 nucleus samples was limited. To alleviate this limitation, SNPs were instead identified using a subset including only the 13 nucleus samples that had a reference genome coverage of 50% or more at 1× (see Supplementary Table 2). This SNP dataset, based on the 13 nucleus samples, was used for the subsequent analysis comparing biallelic SNPs detected in both the whole-organism and the single-nucleus datasets. All 21 individual nucleus samples were later used to estimate the SNP density per nucleus.

### Comparing the detection of polymorphic sites across datasets

The 2 datasets, whole organism and single nuclei, represent distinct ways of capturing intraorganismal genetic variation in *R. irregularis* DAOM197198. To assess the degree of overlap in SNP detection between the 2 datasets, a Venn diagram was generated in R v.4.1.3 (R Core Team 2021) to visualize the intersection of the biallelic SNPs detected across the CDS for both datasets. We then examined the shared SNPs and the dataset-exclusive SNPs (i.e. SNPs identified in 1 dataset but not in the other; hereafter referred to as the “whole-organism exclusive SNPs” and the “single-nucleus exclusive SNPs”) to gain insights into the disparities observed between SNPs identified in the 2 datasets. To do so, we analyzed all SNP sites that were identified in 1 dataset but uncalled in the other by extracting read counts for both reference and alternate alleles in the dataset where the SNPs were not called using the Freebayes v.1.3.2 (Garrison and Marth 2012) naïve variant calling option. In the single-nucleus mapping files, we extracted uncalled sites corresponding to the whole-organism exclusive SNPs and analyzed the sites by applying our nucleus-level hard filter criteria requiring at least 5 reads total and an alternate allele fraction of at least 0.9 within nucleus samples for the alternate allele or an alternate allele fraction of not more than 0.1 for the reference allele. The sites were then scored into the following distinct categories: “potential SNPs” representing sites with at least 1 nucleus supporting an alternate allele; “invariant sites” representing sites where all nuclei with data supported the reference allele; and “sites with missing data” representing sites with only missing data. We then determined if the uncalled sites were initially identified SNPs that failed the nucleus-level and population-level filters or sites that were not initially identified as SNPs by Freebayes (see Supplementary Fig. 2). Similarly, for the whole-organism dataset, we extracted the uncalled sites corresponding to the single-nucleus exclusive SNPs and evaluated their alleles and their read counts. We then categorized the sites as follows: “potential SNPs” representing sites with at least 1 read supporting the alternate allele; “other variants” representing sites with reads for more than 1 alternate allele, multiple nucleotide polymorphisms (MNPs), and indels; or “invariant sites” representing sites that did not have an alternate allele and their reads supported the reference allele. The effect of our population-level filtering criteria was estimated in a second visualization of the intersection of biallelic SNPs detected across the CDS for both datasets without applying the population-level filtering criteria, which required all sites to be supported by data from at least 67% of all the analyzed nucleus samples.

To estimate the likelihood that dataset-exclusive SNPs represent true positives corresponding to false negatives in the other dataset, we compared the proportion of potentially polymorphic sites between the same number of uncalled sites and randomly selected sites in the CDS of both datasets. The expectation for the likelihood test was that if the dataset-exclusive SNPs represent true positives, their corresponding uncalled site would be more likely to score as a potential SNP compared to a randomly selected site in the same dataset. Thus, the proportion of potential SNPs at uncalled sites should be significantly higher than the proportion of potential SNPs observed across randomly sampled sites in the same dataset. For this, a custom script (random_sites_picker_from_bam.py) was used to randomly sample sites to estimate the proportion of potential SNPs as described above for uncalled sites. This process was repeated 10 times. For each dataset, the proportion of potential SNPs in uncalled sites was compared to that in each of the 10 random sets, using a paired chi-square test.

### Evaluation of intraorganismal genetic variation

To evaluate the intraorganismal genetic variation in DAOM197198, we computed the biallelic SNP density across the CDS and the nonrepetitive regions of the reference genome in the whole-organism dataset and separately for 21 single-nucleus samples. In the whole-organism dataset, the biallelic SNP density was determined separately for the CDS and the nonrepetitive regions within the reference genome assembly by separately dividing the number of biallelic SNPs within each assembly fraction by its total size. For the 21 single nuclei, on the other hand, reads covered only part of the genome assembly (see Supplementary Table 2), and SNP density in the CDS and the nonrepetitive regions of the assembly was estimated for each nucleus separately by taking the number of sites with an alternate allele within each assembly fraction that had at least 5× coverage and dividing it by the size of the same fraction with 5× coverage. Thus, for the single nucleus, we considered all sites that were scored as alternate alleles using the nucleus-level hard filter only (at least 5 reads total and an alternate allele fraction of at least 0.9 within nucleus samples) without implementing the population-level filter that required all sites to be supported by data from at least 67% of all the analyzed nucleus samples. We calculated the number of sites with an alternate allele and calculated the average SNP density across the 21 single-nucleus samples. Additionally, an alternate allele accumulation curve and asymptotic SNPs estimates were calculated to estimate the total number of SNPs for the whole organism using the iNEXT package version 2.0.20 (Hsieh *et al*. 2016) with the presence/absence data for all alternate alleles in the CDS fraction across the 21 single-nucleus samples (Supplementary material).

## Results

### Reads from both datasets cover the reference genome assembly

Both short-read sequencing data datasets of *R. irregularis* (DAOM197198) mapped well to the chromosome-level genome assembly of the same strain (Manley *et al*. 2023), with mapping of 99% of the reads on average across the 24 samples in the single-nucleus dataset (see Supplementary Table 2) and in the whole-organism dataset (see Supplementary Table 1). The reads from the whole-organism dataset covered 99.7 and 99.4% of the entire reference genome at read depths of 1× and 5×, respectively (see Supplementary Table 1), while reads from single nuclei, on the other hand, covered on average 50.2 and 29.2% of the reference genome at read depths of 1× and 5×, respectively (see Supplementary Table 2). After removing the 3 nuclei with the least coverage, the numbers for the remaining 21 nuclei increased to 53.7 and 32.1%. Across the best 13 nucleus samples, read coverage reached 60.4 and 38.1% at 1× and 5×, respectively (see Supplementary Table 2). However, when all reads from the 24 nuclei were merged before mapping, their coverage also reached 99% at both 1× and 5× read depths (see Supplementary Table 3). Notably, the whole-organism dataset showed the same high degree of mapping to the other DAOM197198 reference genomes that were evaluated (see Supplementary Methods and Results for details; see Supplementary Table 1). Taken together, these mapping results indicated that both the single-nucleus dataset and the whole-organism dataset offer a comparable and complete representation of the entire reference genome.

### Sampling explains the low concordance in polymorphism discovery between the 2 datasets

Testing the effect of different ploidy settings (1, 2, 5, 10, 15, and 20) on SNP calling using the whole-organism dataset mapped to the DAOM197198 chromosome-level assembly (Manley *et al*. 2023) revealed an improved detection of low-frequency SNPs at higher ploidy settings up to ploidy 10. While requiring extensive compute resources and run times, ploidy settings of above 10 led to only a marginal increase of about 1% in the detected SNPs and did not change the allele frequency distribution (see Supplementary Methods and Results for details; see Supplementary Table 4 and Supplementary Fig. 1). Consequently, we adopted a ploidy setting of 10 for subsequent SNP calling to assess polymorphism detection in both datasets.

**Fig. 1.**
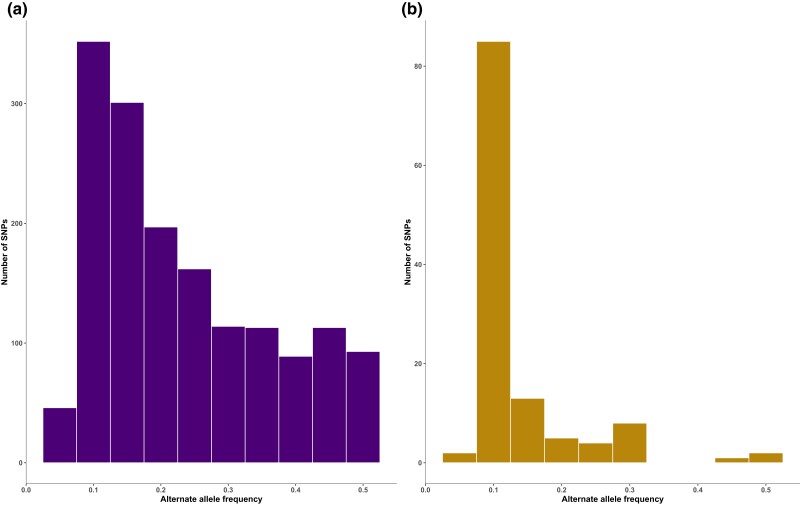
Histogram of AAF distribution in *R. irregularis* DAOM197198 for a) the 1,580 biallelic SNPs in the whole-organism dataset and b) the 120 biallelic SNPs detected across 13 single nuclei.

In the whole-organism dataset, a total of 1,963 SNPs were initially called within the CDS region, before applying any filters. Among these SNPs, 1,608 were biallelic, 146 were triallelic, 157 were tetraallelic, and 52 were pentaallelic (see Supplementary Table 5). After filtering for high-quality SNPs with at least 5 reads supporting the alternate allele, 1,580 biallelic SNPs remained. In the single-nucleus dataset, on the other hand, a total of 12,180 SNPs were initially called in the CDS region. Among these, 11,883 were biallelic, 223 were triallelic, 66 were tetraallelic, and 8 were pentaallelic (see Supplementary Table 5). Because we were cautious to avoid detecting false positives from sequencing errors introduced by MDA, we implemented a stringent nucleus-level filter to ensure that individual nuclei were scored as haploid carrying either a reference or alternate allele and retained only SNP sites with at least 1 nucleus supporting an alternate allele. Further, to allow us to estimate allele frequency, we kept only SNP sites that had data from at least 9 (67%) of the 13 nuclei. Following the application of both nucleus- and population-level filters, only 120 biallelic SNPs remained in the single-nucleus dataset (see Supplementary Table 5). Thus, the vast majority of initially detected SNPs in the single-nucleus dataset did not pass the nucleus-level and the population-level hard filters applied. In the whole-organism dataset, the AAF distribution of the 1,580 biallelic SNPs showed that 649 SNPs (41.1%) were detected at low frequencies (0 ≤ AAF ≤ 0.1667), 542 SNPs (34.3%) were at intermediate frequencies (0.1668 ≤ AAF ≤ 0.3333), and 389 SNPs (24.6%) were of high frequencies (AAF > 0.3333) (Fig. 1a). In contrast, the 120 biallelic SNPs detected across single nuclei were predominantly detected at low frequency, with 99 SNPs (82.5%), and only 18 SNPs (15%) and 3 SNPs (2.5%) occurring in intermediate and high frequencies, respectively (Fig. 1b).

**Fig. 2.**
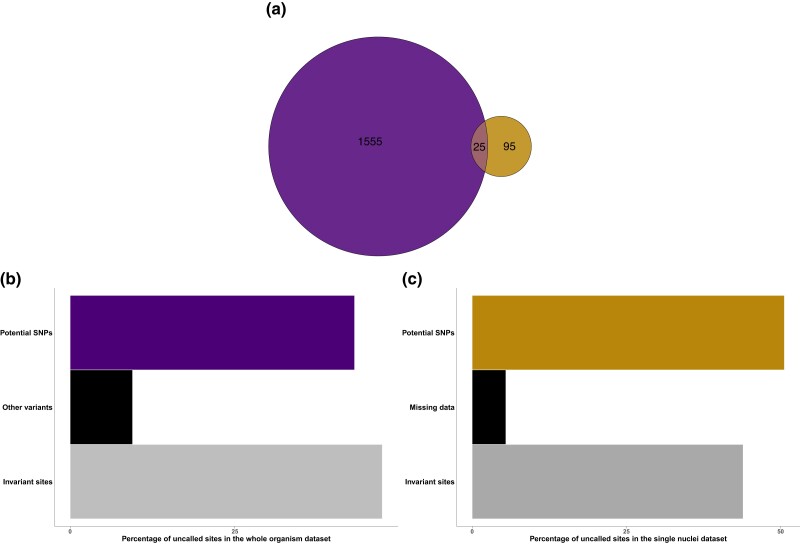
Comparison of SNP detection in *R. irregularis* DAOM197198 using 2 short-read datasets. a) Venn diagram illustrating the number of shared and dataset-exclusive biallelic SNPs between the whole-organism dataset (the left circle) and the single-nucleus dataset (the right circle). b) Bar plot illustrating the distribution across polymorphism categories of 95 uncalled sites in the whole-organism dataset corresponding to the single-nucleus exclusive SNPs. Potential SNPs have at least 1 read supporting an alternate allele. In this dataset, uncalled sites could also be classified as “other variants” for multiallelic sites, MNPs, and indels or “invariant sites” for sites that were invariable for the reference allele. c) Bar plot illustrating the distribution across polymorphism categories of 1,555 uncalled sites in the single-nucleus dataset corresponding to the whole-organism exclusive SNPs. Potential SNPs have at least 1 nucleus supporting an alternate allele. In this dataset, uncalled sites could also be classified as “missing data” for sites with low read counts of less than 5 reads total and/or intermediate alternate allele fractions or “invariant sites” for sites with all available nuclei supporting the reference allele and none supporting the alternate allele.

Remarkably, there was a very low overlap of biallelic SNPs between the 2 datasets. Only 25 SNPs, accounting for only 1.5%, of the total filtered biallelic SNPs across both datasets, were shared between them. Meanwhile, 1,555 and 95 biallelic SNPs were unique to the whole-organism dataset and the single-nucleus dataset, respectively (Fig. 2a). The 25 shared SNPs exhibited a range of AAFs from 0.069 to 0.495, with the majority occurring at lower frequencies. To investigate the low concordance of SNPs identified between the 2 datasets, we examined read counts and alleles of uncalled sites in 1 dataset that corresponded to the uniquely identified SNPs in 1 dataset. Analysis of the allele frequency at 95 uncalled sites in the whole-organism dataset corresponding to the single-nucleus exclusive SNPs revealed that 45 sites (47.3%) were invariant and matched the reference genome alleles (at 86× mean depth) (Fig. 2b). The remaining uncalled sites in the whole-organism dataset consisted of 41 (43.2%) potential SNPs (at least 1 read supporting the alternate allele) and 9 sites (9.5%) classified as other variants (multiallelic SNPs, MNPs, and indels) (Fig. 2b). On the other hand, in the single-nucleus dataset, the 1,555 uncalled sites corresponding to the whole-organism exclusive SNPs consisted of 787 sites (50.6%) that were potential SNPs with at least 1 nucleus supporting an alternate allele. Among the remaining 768 sites, 683 (44%) sites were invariant sites (all nuclei with data supported the reference allele), while 85 sites (5.4%) represented missing data (Fig. 2c).

Interestingly, 635 (40.8%) of the 1,555 uncalled sites were initially called as SNPs in the single-nucleus dataset and were categorized as potential SNPs but were excluded due to insufficient data based on our population-level filtering criteria. An additional 314 (20.2%) sites of the 1,555 uncalled sites were also initially called as SNPs but were excluded because none of the nucleus samples supported an alternate allele based on our nucleus-level filter (see Supplementary Table 6 and Supplementary Fig. 2). The remaining 606 uncalled sites were not initially identified SNPs, and of these, 152 (9.8%) sites were potential SNPs with at least 1 nucleus supporting the alternate allele but did not meet the variant calling quality thresholds, while 454 (29.2%) uncalled sites were considered as invariant sites and sites with missing data based on our hard-filtering criteria (see Supplementary Fig. 2).

**Fig. 3.**
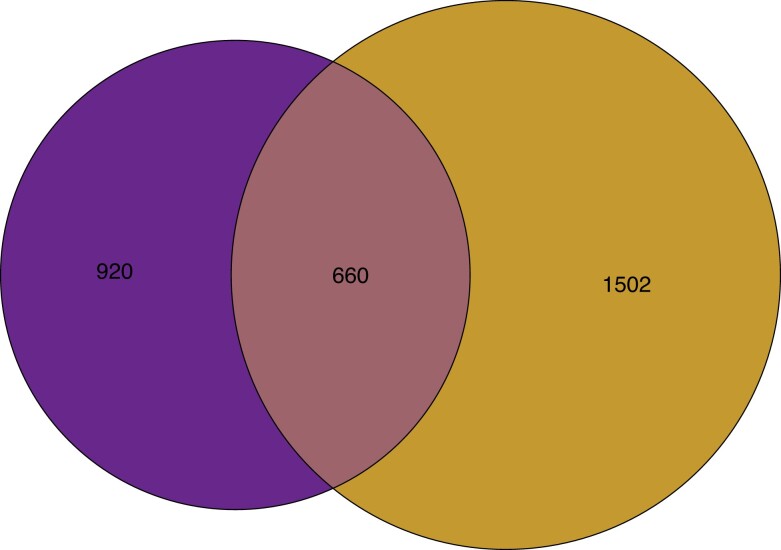
Venn diagram illustrating the number of shared and dataset-exclusive biallelic SNPs between the whole-organism dataset (the left circle) and the single-nucleus dataset (the right circle). In the latter, sites were considered polymorphic as long as at least 1 nucleus supported the alternate allele based on our nucleus-level filter.

Removing the population-level filter increased the overlap of biallelic SNPs identified in the 2 datasets from 25 (1.5%) (Fig. 2a) to 660 SNPs (21.4%) shared between both datasets (Fig. 3). Although there was a 26-fold increase in the overlap when we excluded the population-level filter, concordance in the SNPs identified between the whole-organism and the single-nucleus datasets remained low due to insufficient sampling of nuclei within the whole nucleus population in the organism.

**Fig. 4.**
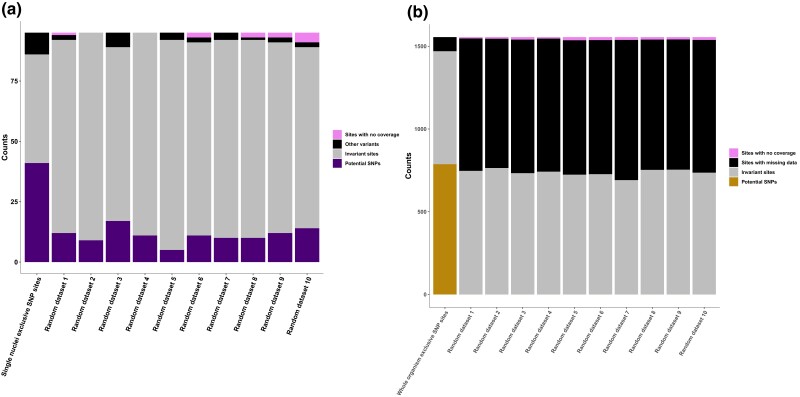
Proportion of sites that scored as potential SNPs, reference, other variants, or sites with no coverage across the uncalled and randomly sampled sites in the CDS of the 2 datasets of *R. irregularis* DAOM197198. a) For the whole-organism dataset, the proportion of potential SNPs (sites with an alternate allele supported by at least 1 read) across 95 uncalled sites corresponding to the single-nucleus exclusive SNPs, compared to 10 sets of random sites in the whole-organism dataset. b) For the single-nucleus dataset, the proportion of potential SNPs (sites with at least 1 nucleus supporting an alternate allele) across 1,555 uncalled sites corresponding to the whole-organism exclusive SNPs, compared to 10 sets of random sites in the single-nucleus dataset.

The discrepancy in SNP detection between the 2 datasets highlights the potential for false negatives in the whole-organism dataset due to failure to detect low-frequency SNPs and in the single-nucleus dataset due to the small fraction of nuclei sampled out of the whole nucleus population in the organism. We tested for false negatives by comparing the proportion of potential SNPs in uncalled sites with the same number of randomly selected sites in the CDS of both datasets. We found that while 43% of the 95 single-nucleus exclusive SNP sites were captured as potential SNPs in the whole-organism dataset (Fig. 2b), this proportion was significantly higher, based on pairwise comparisons to all 10 sets of randomly sampled sites in the same dataset (chi-squared test, *P* < 0.001). Potential SNPs were detected on average in 12% of the random sites (Fig. 4a). While 1 or 2 reads supporting an alternate allele cannot confidently be used to call a SNP in the nucleus population of the whole-organism dataset, it is possible that deeper sequencing would provide further support for these potential low-frequency alternate alleles captured in the single-nucleus dataset. In the single-nucleus dataset, we found that 50.6% of the 1,555 whole-organism exclusive SNP sites had at least 1 nucleus supporting an alternate allele (Fig. 2c), while there were no potential SNPs in the 10 sets of randomly sampled sites (Fig. 4b). Our results show that false negatives are common when calling SNPs in the single-nucleus dataset because many variable sites were not supported by sufficient population-level data (at least 67% of all nucleus samples). By verifying that half of whole-organism exclusive SNPs were indeed supported as alternate alleles in at least 1 nucleus, and as no nuclei supported an alternate allele in the random sites, we conclude that a single nucleus supporting an alternate allele is likely to represent a true variable site that can be used to estimate genetic variation within single nuclei.

### Low levels of intraorganismal genetic variation in *R. irregularis* DAOM197198

To compare estimates of SNP density, we quantified the number of biallelic SNPs present within the entire CDS and the nonrepetitive regions of the reference genome in both the datasets. When calculating SNP density for each of the 21 single nuclei, we considered all sites supported as an alternate allele, based on our hard filters, and divided these by the size of the assembly with 5× read coverage, for both the CDS and the nonrepetitive regions. Overall, the SNP density in the whole-organism dataset was very low, at 0.048 and 0.054 SNPs/kb for the CDS and the nonrepetitive regions, respectively (see Supplementary Table 6). The detected SNP density for individual nuclei was comparable to that of the whole-organism dataset, with per nucleus values ranging from 0.03 to 0.082 SNPs/kb within the CDS and an average density of 0.052 SNPs/kb (see Supplementary Table 7). The nonrepetitive regions had slightly lower values, with SNP densities per nuclei ranging from 0.03 to 0.049 SNPs/kb and an average density of 0.036 SNPs/kb across all 21 nuclei (see Supplementary Table 7). However, across the 21 nuclei, we identified a total of 3,511 unique sites with at least 1 nucleus supporting an alternate allele, and of these, 76% were detected in only 1 nucleus (Supplementary material). The total number of detected SNPs thus depends on the number of sampled nuclei. With a very comprehensive sampling, we estimate that the total number of SNPs in the organism is well above 10,000 sites, based on the asymptotic estimate from the alternate allele accumulation curve (see Supplementary Fig. 3). This suggests that the true SNP density in the organism may be closer to 0.37 SNPs/kb in the CDS, and since most of these variants are rare, they cannot be detected with the current sequencing effort.

## Discussion

In this study, we conducted a comparative analysis of polymorphism discovery in 2 distinct genomic sequence datasets of the AM fungus model species *R. irregularis* strain DAOM197198 for which continuous chromosome-level genome assemblies are available (Yildirir *et al*. 2022; Manley *et al*. 2023). The publicly available datasets include the whole-organism dataset that was generated from pooled spores and hyphae (Chen, Morin, *et al*. 2018) and the single-nucleus dataset that was generated from amplified single nuclei from a single spore (Montoliu-Nerin *et al*. 2021). The 2 datasets represent different ways of sampling variation within the organism and both offer a comparable and complete representation of the entire reference genome with 99% read coverage at 5× (see Supplementary Tables 2 and 3). The identification of genuine polymorphic variants, in particular those at low frequency, is a fundamental challenge in the analysis of population structures and signatures of selection and is influenced by factors like read quality, read depth, and coverage (Everhart *et al*. 2021). The datasets were generated with distinct Illumina sequencing platforms; however, Illumina sequencing technology is renowned for its relatively low error rates, typically in the range of 0.26 to 0.8% (Cheng *et al*. 2023). As such, the likelihood of errors, such as incorrect base calls that could be interpreted as low-frequency polymorphism, occurring during the sequencing process is comparatively low and is not susceptible to consistent errors that could generate false SNPs across multiple samples (Laehnemann *et al*. 2016). Our study examines sampling methods rather than sequencing technology, and our capacity to carry out this comparison was enabled by the availability of a high-quality chromosome-level reference genome assembly (Manley *et al*. 2023). We found that mapping to the recent high-quality chromosome-level reference genomes (Yildirir *et al*. 2022; Manley *et al*. 2023) reduces the estimated SNP density to between half and one-fifth compared to mapping the same dataset to earlier more fragmented genome assemblies of the same strain (Chen, Morin, *et al*. 2018; Montoliu-Nerin *et al*. 2021) (see Supplementary Table 1). We note that the choice of reference genome may have a large impact on genetic diversity estimates.

Detecting low-frequency alleles is particularly important in estimating genetic variation in AM fungi because their genomes are best understood as a population of asexually reproducing haploid nuclei (Jany and Pawlowska 2010; Marleau *et al*. 2011; Kokkoris *et al*. 2020), potentially resulting in multiple alleles per locus and allele frequencies across the full range from 0 to 100 (Wyss *et al*. 2016; Masclaux *et al*. 2019). However, it is common also in AM fungal genomics to use ploidy settings of 1 and 2 in variant calling (Ropars *et al*. 2016; Chen, Mathieu, *et al*. 2018; Chen, Morin, *et al*. 2018; Morin *et al*. 2019; Sahraei *et al*. 2022; van Creij *et al*. 2023). These settings are adapted to haploid or diploid organisms and assume a 1/0 or 50/50 allele frequency distribution, allowing a variant calling software to detect only the 2 most common alleles in a pool or a single sample. A ploidy setting of 10 has been applied in some earlier studies of genetic variation in AM fungi in combination with other variant calling settings for pooled samples (Wyss *et al*. 2016; Savary *et al*. 2018; Masclaux *et al*. 2019). By testing different ploidy settings from 1 to 20, we found that implementing a ploidy of 10 optimized sensitivity toward calling low-frequency SNPs in the whole-organism dataset based on resource use and variant calling sensitivity (see Supplementary Fig. 1 and Supplementary Table 4).

We observed a strikingly low level of overlap in the detected polymorphism with only 25 SNPs shared in both datasets (Fig. 2). When exploring this discrepancy, we found that in the whole-organism dataset, a substantial proportion (47.3%) of the 95 uncalled sites corresponding to the single-nucleus exclusive SNPs were indeed invariant and supported the reference allele (Fig. 2b). In the single-nucleus dataset, on the other hand, 44% of the 1,555 uncalled sites corresponding to the whole-organism exclusive SNPs were invariant (Fig. 2c). To understand this discrepancy, it is important to remember that most alternate alleles occur at a low frequency and that the 2 datasets differ in their ability to detect low-frequency alleles present only in a fraction of all nuclei in the organism (Fig. 1). In the whole-organism dataset, we scored alternate alleles only if they were supported by at least 5 reads. With an average sequencing depth of 86× (see Supplementary Table 1), AAFs below 5% are rarely detected (Fig. 1a). It is known that low-frequency variants are generally challenging to detect in sequence data from pooled samples, which mainly allows for the discovery of common variants of moderate to high frequencies (Fuentes-Pardo and Ruzzante 2017). This is because pooled samples represent an average of allele frequencies across multiple individuals potentially masking low-frequency variants present in individual samples (Raineri *et al*. 2012). It has also been demonstrated that rare variants potentially occurring in only a small fraction of all nuclei of the multinucleate *R. irregularis* are not easily detected (Masclaux *et al*. 2019; van Creij *et al*. 2023). In the single-nucleus dataset on the other hand, the detection of rare variants is subject to chance during the nucleus sorting (Montoliu-Nerin *et al*. 2020), and, in this dataset, most alternate alleles are detected at low frequencies (Fig. 1b) often found only in 1 nucleus sample. Hence, it is not surprising that most of the variants detected in the single-nucleus dataset were uncalled in the whole-organism dataset since these SNPs likely occur in only a fraction of all nuclei within the analyzed strain. It is important to note that although both datasets are generated from the same strain, *R. irregularis* DAOM197198, originally isolated in Pont Rouge, Canada (Stockinger *et al*. 2009), genetic differences between the sequenced samples are plausible. Distinct mutations may have accumulated in the cultures, as the whole-organism dataset was generated from a culture maintained at Agronutrition (Labège, France) (Chen, Morin, *et al*. 2018) while the single-nucleus dataset was generated from a spore purchased from AAFC, Canada (Montoliu-Nerin *et al*. 2021). Genetic differences have been observed between individual AM fungal spores, single spore lines, and even among culture batches of the same spore lines in previous studies (Ehinger *et al*. 2012; Masclaux *et al*. 2019; Robbins *et al*. 2021). It was also recently demonstrated that progeny spores of the *R. irregularis* C3 strain can undergo random loss of genetic variation compared to their parental single spore lines even when cultured under the same conditions (van Creij *et al*. 2023). Thus, the genetic composition of a single spore may not fully represent the entire genetic variation of the organism (van Creij *et al*. 2023). Thus, it is plausible that part of the discrepancy in SNP detection between the 2 datasets is due to actual variation between their biological materials analyzed.

Our main concern when working with the single-nucleus dataset was the potential for false positives during SNP calling due to sequence errors introduced by MDA of single-nucleus samples (Auxier and Bazzicalupo 2019). Thus, to minimize false positives in this dataset and to account for the biology of our study organism, we implemented stringent filters, scoring alleles only if they were supported by at least 5 reads with an allele fraction of 0.1 or below for reference and 0.9 or above for alternate within each nucleus. Before scoring a site as variable, we also required it to be supported by at least 67% of the nucleus samples. The population-level filter significantly reduced the number of identified biallelic SNPs in the single-nucleus dataset (see Supplementary Table 5). Interestingly, we found that 40.8% of the uncalled sites in the single-nucleus dataset corresponding to the 1,555 whole-organism exclusive SNPs supported an alternate allele in at least 1 nucleus but failed our population-level filter. Further, an additional 9.8% did not pass the Freebayes quality filters (see Supplementary Fig. 2), likely due to uneven coverage and noise from the MDA in the single-nucleus dataset. Sufficient sampling and sequencing depth are necessary to detect more low-frequency variants and to better distinguish true rare variants from potential sequencing errors (Fuentes-Pardo and Ruzzante 2017). Improved methods for high-coverage sequencing of single nuclei would effectively address the issue that a fraction (9.8%) of uncalled sites in the single-nucleus dataset did not pass the basic quality filters in Freebayes. We conclude that, overall, the population-level hard-filtering criteria were the primary reason for the initially observed discordance between the whole-organism dataset and the single-nucleus dataset (Fig. 2). Similar stringent hard-filtering criteria have been implemented in other previous studies utilizing single-nucleus sequencing data to investigate diversity in AM fungi, leading to significant reductions of approximately 75 to 90% in the final number of sites retained for analyses compared to unfiltered data (Chen, Mathieu, *et al*. 2018; Auxier and Bazzicalupo 2019; Chen *et al*. 2020; van Creij *et al*. 2023). However, the stringent hard-filtering criteria did not confound their observations of signatures of potential recombination events (Chen *et al*. 2020; van Creij *et al*. 2023). By comparing SNP detection in the 2 datasets, we firmly established that alternate alleles detected in single nuclei, after stringent filtering at the nucleus level, are likely to represent true variants in the organism. Sequencing individual nuclei enables haplotype characterization in the nucleus population, which is not achievable with pooled samples like the whole-organism dataset. While the latter facilitates the detection of common variants, it comes at the cost of losing individual haplotype information, making the detection of low-frequency alleles more difficult (Cutler and Jensen 2010).

Both datasets confirm earlier observations of very low levels of intraorganismal genetic variation in *R. irregularis* DAOM197198 (Tisserant *et al*. 2013; Lin *et al*. 2014; Morin *et al*. 2019). We estimated SNP densities within the CDS regions to an average of 0.052 SNPs/kb across nuclei and 0.048 SNPs/kb in the whole-organism dataset (see Supplementary Tables 6 and 7), notably lower than previously reported estimates of 0.23–0.43 SNPs/kb for the DAOM197198 strain (Tisserant *et al*. 2013; Lin *et al*. 2014; Morin *et al*. 2019). We attribute this difference mainly to the high quality and completeness of the reference genome used (Manley *et al*. 2023), as more fragmented assemblies used for read mapping resulted in a higher number of detected variants (see Supplementary Table 1). In the single-nucleus dataset, most SNPs are rare across nuclei (Fig. 1b), and the estimated asymptotic SNP density in the whole organism may be closer to 0.37 SNPs/kb in the CDS (see Supplementary Fig. 2), which is significantly higher than what can be detected using pooled samples at the current sequencing depth. However, the number of single nuclei that would have to be sequenced individually to characterize all unique haplotypes in the strain may not be affordable or feasible at this point, given that 76% of the alternate alleles were detected in only a single nucleus, and we expected most variants to only occur in a diminishingly small fraction of the nuclei. The pattern of mostly rare variants accumulating in single nuclei aligns with our conceptual understanding that AM fungal strains are genetically organized like a population of independently reproducing haploid individuals (Jany and Pawlowska 2010; Marleau *et al*. 2011; Manley *et al*. 2023). AM fungi propagate predominantly as clones (van Creij *et al*. 2023) with no known single-nucleus stage in their life cycle (Bonfante and Genre 2010; Sędzielewska *et al*. 2011; Kokkoris *et al*. 2020). Having large populations of nuclei with asynchronized division within their multinucleate mycelia in combination with their mode of sporulation has been suggested to be an adaptation that allows for selection to act on nuclei within the mycelia to purge deleterious mutations (Jany and Pawlowska 2010). Purifying selection may be an important mechanism by which *R. irregularis*, and possibly other AM fungi purge deleterious mutations arising in single nuclei.

In summary, we assessed the distributions of allele frequencies within whole-genome sequence data of the *R. irregularis* DAOM197198 strain generated from pooled spores and mycelia and amplified single nuclei from a single spore. Our findings highlight sampling limitations for the detection of low-frequency variants, as evidenced by variations in allele frequencies of detected SNPs in the 2 datasets. Our data demonstrate that most variants are present only in a small fraction of the nuclei potentially limiting their detection in pooled samples like the whole-organism dataset. We estimate that the actual SNP density across all nuclei could be up to 8 times higher than that captured in our data, but extensive sequencing would be necessary to confirm this. Overall, our study demonstrates the applicability of single-nucleus data for polymorphism discovery that has potential not only in AM fungi but also in other nonmodel asexually reproducing organisms that may be difficult to sequence due to their complicated biology.

## Supplementary Material

jkae074_Supplementary_Data

## Data Availability

The raw reads for the single-nucleus dataset used in this study are available at ENA with the project number PRJEB45340. The raw reads for the whole-organism dataset used in this study were published by Chen *et al*. (2018) and provided by Francis Martin (INRA, France). Custom scripts and data are available at https://github.com/drowl001/Comparative_polymorphism_discovery. Supplemental material available at G3 online.
