# Supplementary material for: Detection of rare variants among nuclei populating the arbuscular mycorrhizal fungal model species Rhizophagus irregularis DAOM197198
Source: G3 (Bethesda). 2024 Apr 24;14(6):jkae074. doi: 10.1093/g3journal/jkae074 (PMC11152072; doi:10.1093/g3journal/jkae074)
Supplement: jkae074_Supplementary_Data [file jkae074_supplementary_data.zip › File_S1_G3-2024-405012.docx]

**Supplementary Tables and Figures**

Manyara *et al.* Detection of rare variants among nuclei populating the arbuscular mycorrhizal (AM) fungal model species *Rhizophagus irregularis* DAOM197198.

**Table S1.** Summary statistics for the *R. irregularis* DAOM197198 genome assemblies, whole organism dataset mapping statistics, and counts of bi-allelic SNPs.

| **Reference Assembly** | **Manley et al. (2023)** | **Yildirir et al. (2022)** | **Montoliu-nerin et al. (2021)** | **Chen, Morin, et al. (2018)** |
| --- | --- | --- | --- | --- |
| Assembly level | Chromosome | Chromosome | Contig | Scaffold |
| Number of contigs | 42 | 107 | 15,939 | 5,810 |
| Number of scaffolds | 32 | 33 | - | 1,123 |
| Assembly size (bp) | 146,773,001 | 147,209,168 | 116,452,263 | 136,807,476 |
| Number of genes | 30,209 | 26,634 | 23,258 | 26,183 |
| Percentage of reads aligned to the genome assembly | 98.74 | 98.75 | 98.97 | 98.67 |
| Percentage of the genome assembly covered by reads (1X) | 99.74 | 98.94 | 99.94 | 94.81 |
| Percentage of the genome assembly covered by reads (5X) | 99.60 | 99.91 | 99.89 | 94.64 |
| Mean sequencing depth | 86.27 | 186.65 | 107.87 | 91.92 |
| Counts of SNPs in only the CDS fraction of the genome | 1,580 | 660 | 33,882 | 21,032 |
| SNP density in only the CDS fraction of the genome | 0.048 | 0.022 | 0.790 | 0.819 |

**Table S2.** Mapping statistics for the 24 nuclei of the *R. irregularis* DAOM197198 single nuclei dataset when mapped to the DAOM197198 chromosome-level assembly 2.

| **Nucleus** | **Percentage of reads aligned to the genome assembly** | **Percentage of the genome assembly covered by reads (1X)** | **Percentage of the genome assembly covered by reads (5X)** | **Mean sequencing depth** |
| --- | --- | --- | --- | --- |
| ****1** | 99.41 | 50.83 | 29.24 | 26.28 |
| **^+^2** | 99.54 | 24.77 | 8.94 | 26.22 |
| ****3** | 99.62 | 52.08 | 29.67 | 24.74 |
| ****4** | 99.56 | 54.7 | 32.34 | 23.41 |
| ****5** | 99.54 | 50.37 | 27.49 | 32.19 |
| ****6** | 99.43 | 53.08 | 30.79 | 25.58 |
| ****7** | 99.62 | 51.85 | 29.38 | 34.31 |
| **8** | 93.71 | 39.58 | 19.82 | 23.47 |
| ****9** | 99.53 | 66.15 | 42.84 | 31.16 |
| ****10** | 99.62 | 66.3 | 43.69 | 36.94 |
| ****11** | 99.64 | 67.86 | 44.89 | 27.65 |
| **^+^12** | 99.58 | 19.46 | 3.09 | 28.52 |
| ****13** | 99.61 | 63.74 | 40.09 | 46.26 |
| ****14** | 92.68 | 53.91 | 30.73 | 38.44 |
| ****15** | 99.51 | 88.85 | 69.92 | 31.16 |
| **16** | 99.12 | 46.86 | 27.19 | 27.96 |
| **17** | 99 | 47.3 | 25.57 | 30.12 |
| **18** | 99.66 | 40.79 | 20.47 | 27.64 |
| ****19** | 99.56 | 65.52 | 43.81 | 34.04 |
| **20** | 99.51 | 48.23 | 25.99 | 31.81 |
| **21** | 99.65 | 37.49 | 18.49 | 29.43 |
| **22** | 99.65 | 43.82 | 22.91 | 35.44 |
| **^+^23** | 99.67 | 32.17 | 15.24 | 30.61 |
| **24** | 99.39 | 39.24 | 19.03 | 27.7 |
| **Average (N=24)** | **98.99** | **50.21** | **29.23** | **30.46** |
| **Average (N=21)** | **98.91** | **53.74** | **32.11** | **30.75** |
| **Average (N=13)** | **99.02** | **60.4** | **38.07** | **31.7** |

**^+^**  The three nuclei excluded from further analysis

* The 13 nuclei with best coverage

**Table S3.** Mapping statistics for the *R. irregularis* DAOM197198 merged 24 single nuclei dataset when mapped to the DAOM197198 chromosome assembly 2.

| **Dataset** | **Percentage of reads aligned to the genome assembly** | **Percentage of the genome assembly covered by reads (1X)** | **Percentage of the genome assembly covered by reads (5X)** |
| --- | --- | --- | --- |
| **Merged 24 nuclei** | 98.94 | 99.71 | 99.43 |

**Table S4.** Summary of the counts of bi-allelic SNPs only in the CDS regions of the *R. irregularis* DAOM197198 whole organism dataset alignment to the DAOM197198 chromosome assembly 2 at different ploidy settings.

| **Ploidy** | **Counts of SNPs in only the CDS fraction of the genome** | **Counts of low, intermediate and high-frequency SNPs** |
| --- | --- | --- |
| Ploidy 1 | 612 | 520/ 17/ 75 |
| Ploidy 2 | 877 | 8/ 478/ 391 |
| Ploidy 5 | 1,410 | 477/ 543/ 390 |
| Ploidy 10 | 1,580 | 649/ 542/ 389 |
| Ploidy 15 | 1,599 | 668/ 542/ 389 |
| Ploidy 20 | 1,590 | 659/ 542/ 389 |

**Table S5.** Summary of the counts of bi-allelic and multi-allelic SNPs only in the CDS regions of the *R. irregularis* DAOM197198 whole organism dataset and *R. irregularis* DAOM197198 13 single nucleus dataset when mapped to the DAOM197198 chromosome assembly 2.

| **Dataset** | **2 alleles** | **3 alleles** | **4 alleles** | **>4 alleles** |
| --- | --- | --- | --- | --- |
| Whole organism dataset | 1,608 | 146 | 157 | 52 |
| After filtering | 1,580 | - | - | - |
| 13 single nucleus dataset | 11,883 | 223 | 66 | 8 |
| After nucleus- and population-level filtering | 2,162 | - | - | - |
| After nucleus-level filtering | 120 | - | - | - |

**Table S6.** Summary of the SNP density within the CDS and the non-repeat fractions (at a read depth of 5X) of the *R. irregularis* DAOM197198 whole organism dataset

| **Reference Assembly** | **Number of SNPs** | **SNPs/kb** |
| --- | --- | --- |
| The CDS fraction of the genome | 1,580 | 0.048 |
| The non-repetitive fraction of the genome | 4,399 | 0.054 |

**Table S7.** Summary of the SNP density within the CDS and the non-repeat fractions (at a read depth of 5X) of the *R. irregularis* DAOM19719821 single nuclei.

| **CDS fraction Non-repeat fraction** | | | | |
| --- | --- | --- | --- | --- |
| **Nuclei** | **Number of SNPs** | **SNPs/kb** | **Number of SNPs** | **SNPs/kb** |
| **1** | 404 | 0.048 | 908 | 0.035 |
| **3** | 359 | 0.042 | 780 | 0.030 |
| **4** | 395 | 0.041 | 924 | 0.032 |
| **5** | 397 | 0.053 | 964 | 0.039 |
| **6** | 386 | 0.043 | 888 | 0.032 |
| **7** | 457 | 0.055 | 1,018 | 0.039 |
| **8** | 362 | 0.065 | 848 | 0.048 |
| **9** | 559 | 0.045 | 1,172 | 0.031 |
| **10** | 454 | 0.036 | 1,084 | 0.028 |
| **11** | 567 | 0.043 | 1,152 | 0.029 |
| **13** | 429 | 0.038 | 1,072 | 0.030 |
| **14** | 499 | 0.057 | 1,064 | 0.039 |
| **15** | 646 | 0.030 | 1,539 | 0.025 |
| **16** | 412 | 0.049 | 865 | 0.036 |
| **17** | 388 | 0.054 | 779 | 0.034 |
| **18** | 475 | 0.082 | 881 | 0.049 |
| **19** | 646 | 0.052 | 1,294 | 0.033 |
| **20** | 385 | 0.052 | 860 | 0.037 |
| **21** | 355 | 0.068 | 748 | 0.046 |
| **22** | 438 | 0.067 | 855 | 0.042 |
| **24** | 422 | 0.073 | 801 | 0.047 |
| **Average** | **449** | **0.052** | **976** | **0.036** |





**Figure S1**. Histograms of alternate allele frequency distributions of the bi-allelic SNPs in the CDS regions of the *R. irregularis* DAOM197198 whole organism dataset alignment to the DAOM197198 chromosome-level assembly 2 at different ploidy settings of 1, 2, 5, 10, 15, and 20, respectively.


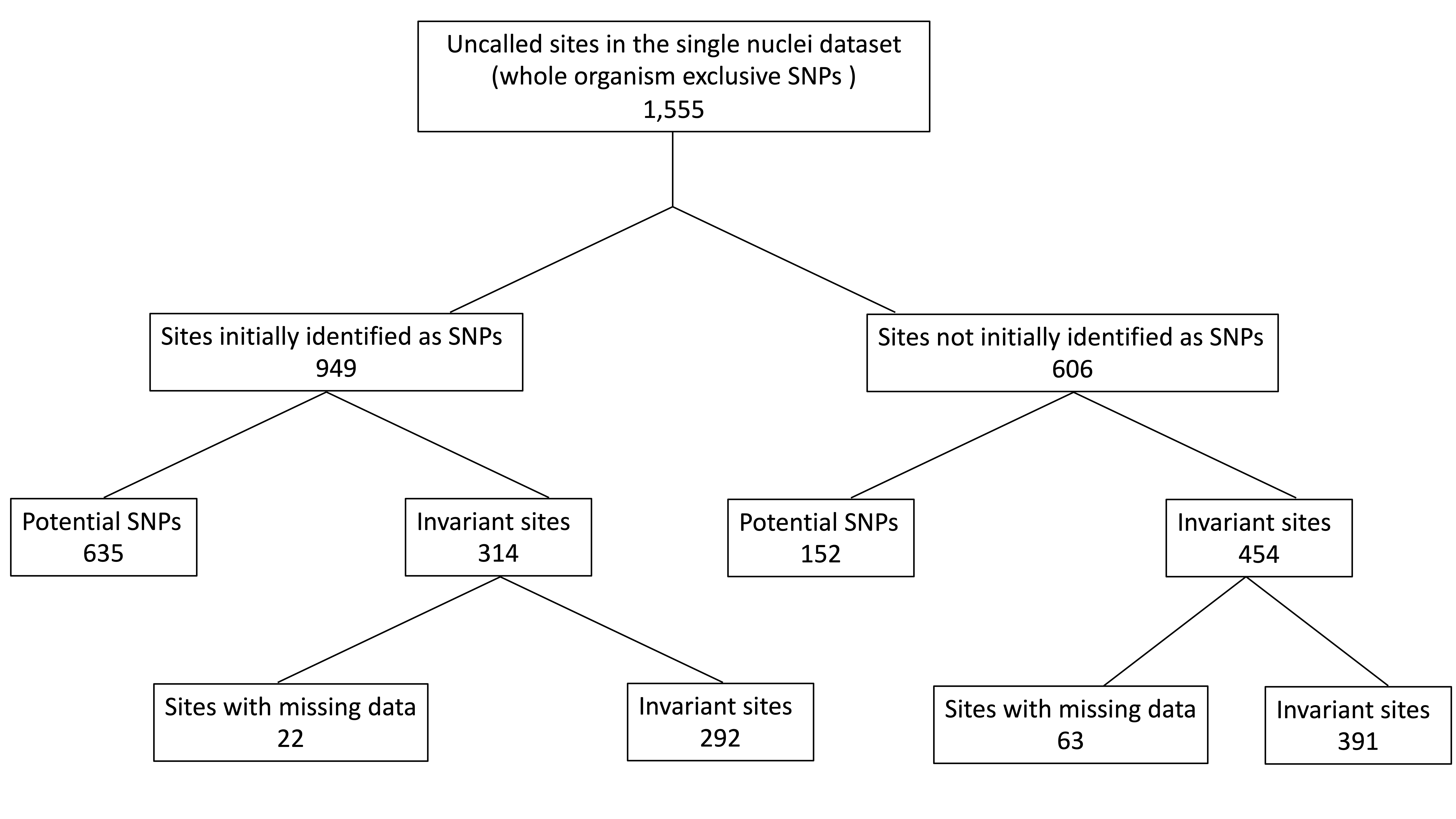


**Figure S2**. Flow chart showing broad categorization of the 1,555 uncalled sites in the single nuclei dataset corresponding to the whole organism exclusive SNPs. The sites were either initially identified as SNPs in the single nuclei dataset but subsequently excluded after failing our nucleus-level and population-level filters, or not initially identified as SNPs. ‘Potential SNPs’ are sites with at least one nucleus supporting an alternate allele while ‘Invariant sites’ are sites with all available nuclei supporting the reference allele and none supporting the alternate allele. ‘Sites with missing data’ are sites with low read counts of less than 5 reads total and/or intermediate alternate allele fractions.


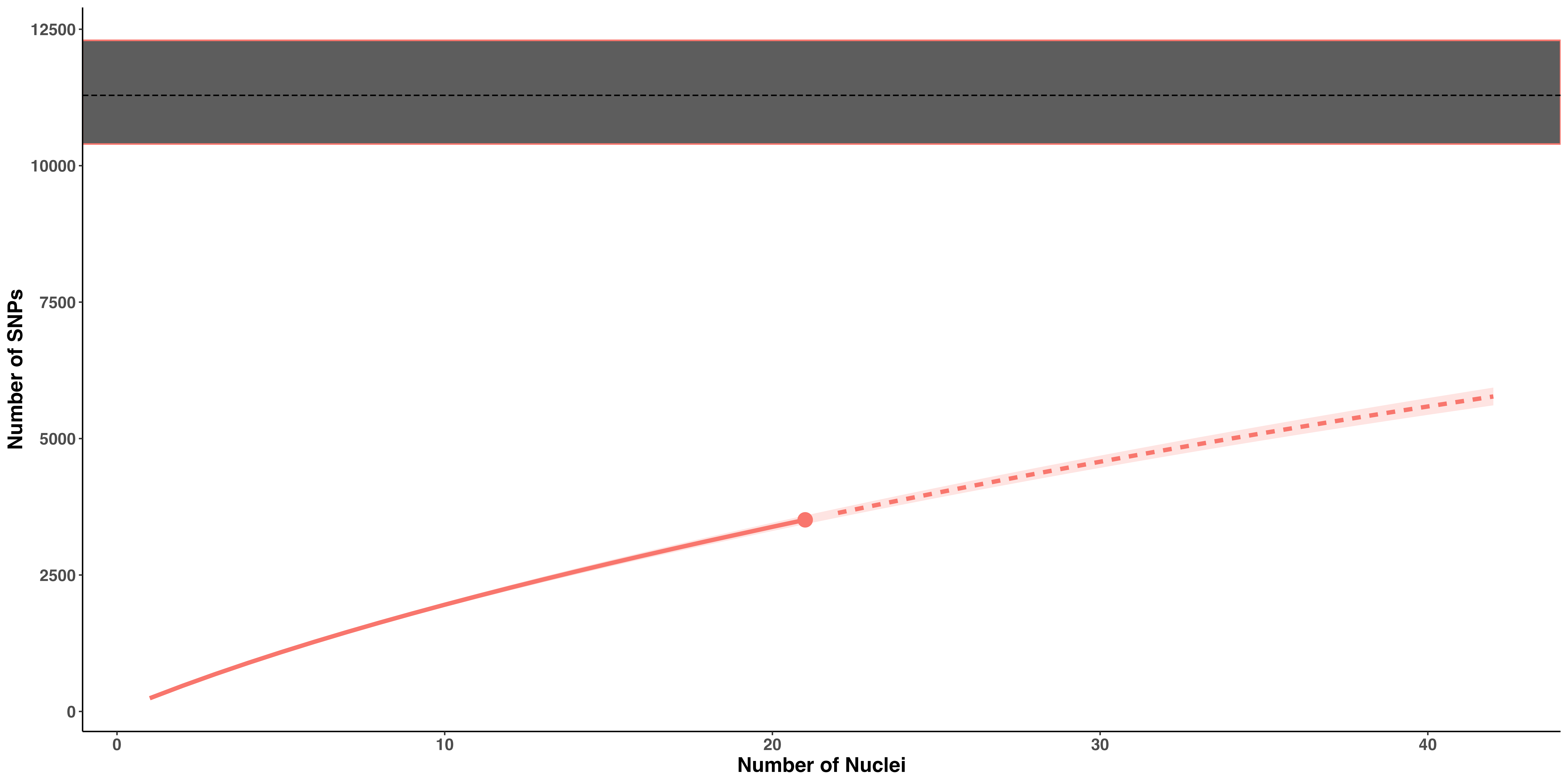


**Figure S3**. Alternate allele accumulation curve with 95% confidence intervals based on the presence/absence data of all the alternate alleles in the CDS fraction of the 21 single nuclei samples. The solid line corresponds to interpolation and the dashed line corresponds to the extrapolation curve up to the base sample size of 42 nuclei which corresponds to double the reference sample size of 21 nuclei. The 95% confidence intervals were obtained by a bootstrap method based on 50 replications.
