# Supplementary material for: Detection of rare variants among nuclei populating the arbuscular mycorrhizal fungal model species Rhizophagus irregularis DAOM197198
Source: G3 (Bethesda). 2024 Apr 24;14(6):jkae074. doi: 10.1093/g3journal/jkae074 (PMC11152072; doi:10.1093/g3journal/jkae074)
Supplement: jkae074_Supplementary_Data [file jkae074_supplementary_data.zip › Supplementary_Methods_and_Results_G3-2024-405012.docx]

**Supplementary Methods and Results**

Manyara *et al.* Detection of rare variants among nuclei populating the arbuscular mycorrhizal (AM) fungal model species *Rhizophagus irregularis* DAOM197198.

**Supplementary Methods**

**Read mapping**

To evaluate the impact of the reference assembly on the estimation of intra-organismal genetic variation, we mapped only the whole organism data to four reference genomes namely; the DAOM197198 chromosome-level reference genome assembly recently published by Manley and colleagues (2023) (hereafter referred to as DAOM197198 chromosome-level assembly 2), the DAOM197198 chromosome-level assembly published by Yildirir and colleagues (2022) (hereafter referred to as DAOM197198 chromosome-level assembly 1), the DAOM197198 assembly generated by Montoliu-Nerin and colleagues (2021), and the DAOM197198 V2.1 published by Chen and colleagues (2018) (See Table S1 in File S1). The DAOM197198 chromosome-level assembly 2 was generated from Nanopore read data (prepared from *Rhizophagus irregularis* DAOM197198 pooled spores), scaffolded with Hi-C read data generated in the study by Yildirir and colleagues (2022), and polished with Illumina read data generated in the study by Maeda and colleagues (2018) (Manley et al. 2023). The DAOM197198 chromosome-level assembly 1 was generated from Nanopore read data, scaffolded with Hi-C read data (both prepared from pooled mycelium and spores), and polished with Illumina read data generated in the study by Chen and colleagues (2018) (Yildirir et al. 2022). The DAOM197198 assembly from the study by Montoliu-Nerin and colleagues (2021) was generated from Illumina reads prepared from 24 amplified and individually sequenced nuclei (Montoliu-Nerin et al. 2021). The DAOM197198 V2.1 was generated from Illumina reads prepared from pooled mycelium and spores (Chen et al. 2018).

**Variant calling**

To evaluate the effect of the ploidy settings on the detection and frequency distribution of SNPs, different ploidy settings of 1, 2, 5, 10, 15, and 20 were first explored using only the whole organism dataset mapped to DAOM197198 chromosome-level assembly 2 (See Table S4 in File S1).

**Supplementary Results**

**Read mapping**

The mapping rates for the whole organism dataset to the four reference genomes were high and comparable at averages of 98.7%, 98.8%, 99%, and 98.7% to the DAOM197198 chromosome-level assembly 2, the DAOM197198 chromosome-level assembly 1, the DAOM197198 assembly, and the DAOM197198 V2.1, respectively (See Table S1 in File S1). The reads from the whole organism dataset covered the two chromosome-level assemblies and the DAOM197198 assembly extensively, with an average of 99% coverage at both read depths of 1X and 5X. The coverage for the DAOM197198 V2.1 was, however, comparatively lower at an average of 94% for both 1X and 5X read depths (See Table S1 in File S1).

**Variant calling**

To assess the impact of ploidy settings on the detection and frequency distribution of SNPs, we tested ploidy settings of 1, 2, 5, 10, 15, and 20 in only the whole organism dataset mapped to the DAOM197198 chromosome assembly 2 (See Table S4 in File S1). The number of bi-allelic SNPs detected within the CDS regions markedly increased with higher ploidy settings of 2, 5, and 10 but showed comparable numbers at higher settings of 10, 15, and 20 (See Table S4 in File S1). Similarly, a larger proportion of low-frequency SNPs (0 ≤ AAF ≤ 0.1667) were detected at ploidy settings of 1 and higher settings of 10, 15, and 20, compared to intermediate (0.1668 ≤ AAF ≤ 0.3333) and high-frequency SNPs (AAF > 0.3333) at these ploidy settings (See Table S4; Figure S1 in File S1).

To assess the impact of the reference assembly on the estimation of intra-organismal genetic variation, we called SNPs with a ploidy setting of 10 in only the whole organism dataset mapped to the four different reference assemblies evaluated (See Table S1 in File S1). The number of bi-allelic SNPs detected varied highly with 1,580, 660, 33,882, and 21,032 filtered bi-allelic SNPs detected in the CDS regions of DAOM197198 chromosome-level assembly 2, DAOM197198 chromosome-level assembly 1, DAOM197198 V2.1, and the DAOM197198 assembly, respectively (See Table S1 in File S1). This corresponded to a SNP density of 0.048, 0.022, 0.790, and 0.819 for the DAOM197198 chromosome-level assembly 2, DAOM197198 chromosome-level assembly 1, DAOM197198 V2.1, and the DAOM197198 assembly, respectively (See Table S1 in File S1).

**References**

Chen, Eric, Emmanuelle Morin, Denis Beaudet, Jessica Noel, Gokalp Yildirir, Steve Ndikumana, Philippe Charron, et al. 2018. “High Intraspecific Genome Diversity in the Model Arbuscular Mycorrhizal Symbiont Rhizophagus Irregularis.” *New Phytologist* 220 (4): 1161–71. https://doi.org/10.1111/NPH.14989.

Manley, Bethan F., Jaruwatana S. Lotharukpong, Josué Barrera-Redondo, Theo Llewellyn, Gokalp Yildirir, Jana Sperschneider, Nicolas Corradi, Uta Paszkowski, Eric A. Miska, and Alexandra Dallaire. 2023. “A Highly Contiguous Genome Assembly Reveals Sources of Genomic Novelty in the Symbiotic Fungus Rhizophagus Irregularis.” *G3 Genes|Genomes|Genetics* 13 (6): 77. https://doi.org/10.1093/G3JOURNAL/JKAD077.

Montoliu-Nerin, Merce, Marisol Sánchez-García, Claudia Bergin, Verena Esther Kutschera, Hanna Johannesson, James D. Bever, and Anna Rosling. 2021. “In-Depth Phylogenomic Analysis of Arbuscular Mycorrhizal Fungi Based on a Comprehensive Set of de Novo Genome Assemblies.” *Frontiers in Fungal Biology* 2 (September): 53. https://doi.org/10.3389/ffunb.2021.716385.

Yildirir, Gökalp, Jana Sperschneider, Mathu Malar C, Eric C.H. Chen, Wataru Iwasaki, Calvin Cornell, and Nicolas Corradi. 2022. “Long Reads and Hi-C Sequencing Illuminate the Two-Compartment Genome of the Model Arbuscular Mycorrhizal Symbiont Rhizophagus Irregularis.” *New Phytologist* 233 (3): 1097–1107. https://doi.org/10.1111/NPH.17842.
